# Comparison of the Diagnostic Accuracy of an AI-Based System for Dental Caries Detection and Clinical Evaluation Conducted by Dentists

**DOI:** 10.3390/jcm14051566

**Published:** 2025-02-26

**Authors:** Jakub Kwiatek, Marta Leśna, Wiktor Piskórz, Justyna Kaczewiak

**Affiliations:** Kwiatek Dental Clinic, Kordeckiego 22, 60-144 Poznań, Poland; jakubkwiatek@klinikakwiatek.pl (J.K.); piskorzwiktor@gmail.com (W.P.); justynakaczewiak1@gmail.com (J.K.)

**Keywords:** dental caries, dental diagnostics, artificial intelligence (AI), Diagnocat, machine learning, panoramic radiographs, blind study

## Abstract

**Background/Objectives:** Artificial intelligence (AI)-based software is increasingly used for radiographic analysis in dentistry. This study aimed to evaluate the diagnostic accuracy of an AI-powered radiographic analysis system, using Diagnocat (DGNCT LLC, Miami, FL, USA) as an example, compared with clinical evaluations performed by three experienced dentists. The assessment focused on primary caries detection and the total number of primary and secondary caries based on panoramic radiographs (OPGs). **Methods:** Three dentists with similar expertise independently classified teeth for treatment using only panoramic radiographs and their clinical knowledge. The study was conducted under single-blind conditions, where clinicians were unaware that their diagnoses would be compared to the AI system’s analysis. **Results:** The AI system’s agreement with human evaluations varied depending on tooth location, patient age, and gender. The lowest agreement was observed for premolars, likely due to limitations of 2D imaging, while higher accuracy was found for molars and incisors, particularly in younger patients. The system showed limitations in detecting occlusal, labial, and lingual caries. **Conclusions:** AI-assisted radiographic analysis has the potential to enhance diagnostic efficiency and automation in dentistry. However, its accuracy is influenced by tooth location and imaging modality. Further research is needed to explore the benefits of integrating AI with 3D imaging techniques to improve diagnostic reliability.

## 1. Introduction

Since the introduction of ChatGPT, artificial intelligence (AI) and machine learning have become key topics of research across various scientific disciplines.

The potential application of intelligent algorithms to assist or even replace human decision-making in data, image, and event analysis presents both a challenge and an area of growing interest in modern medicine [1].

This issue is analyzed from two perspectives: first, the responsibility for patient health, medical regulations, and the accountability for AI-based diagnoses; second, the vast amount of health-related data, which may lead to information overload and pose challenges for specialists in processing excessive data [2].

From a clinical perspective, the amount of input data about a patient often exceeds the analytical capacity of a physician specializing in a single field. In dentistry alone, this includes radiological examinations, laboratory tests, photographs, medical history (subjective examination), and physical findings (objective examination of teeth, oral mucosa, craniofacial musculature, and lymph nodes). These data are often overlooked when they do not directly relate to the patient’s presenting condition or are not subjected to further analysis [3]. In this context, artificial intelligence can be a valuable tool for integrating diverse data sources [4].

Current research into the utility of AI systems compares the sensitivity and specificity of diagnoses made by AI with those made by clinicians. This comparison is typically based on analyses of the same panoramic radiograph (or CBCT) performed by a dentist and the AI system [5,6,7,8,9]. The methodology of these studies can be analyzed in analogy to the distinction between in vivo (within the organism) and in vitro (laboratory-based) research. Two main areas of potential error emerge from the study design assumptions.

First, analyzing an image without the presence of a patient prompts the clinician to examine every detail meticulously, documenting all structures with precision. This scenario differs significantly from clinical practice, where a patient presents with specific complaints requiring immediate diagnosis. In such cases, the clinician naturally focuses on the primary issue, narrowing the scope of their analysis. It should be considered that diagnostic errors, which represent a significant issue in dentistry [10], predominantly arise in real clinical settings. Therefore, to effectively analyze the impact of AI systems on diagnostics, it is essential to replicate the actual conditions under which diagnostic decisions are made.

Second, in real clinical settings, dentists do not rely solely on radiographic images. Physical examination remains an essential component of the consultation, providing critical information for accurate diagnosis.

Additionally, some studies compare the diagnostic performance of AI systems to that of dental radiologists under conditions without time constraints, which is a stark contrast to typical dental consultations.

In many studies, image analysis is conducted in non-clinical (in vitro) settings, where patients are absent and no therapeutic decisions are required. These analyses are performed in controlled environments with unlimited review time, making them significantly different from real-world clinical conditions. In the literature review we conducted, the authors primarily focused on comparing the results of image analysis performed by AI systems with those obtained by human clinicians. However, these studies were often conducted in isolation from complete clinical settings. Zadrożny et al., in their study, evaluated 30 panoramic radiographs using the automatic AI analysis provided by Diagnocat (DGNCT LLC, Miami, FL, USA), as well as manual assessments conducted by three dentists with varying levels of experience and subsequently compared the agreement between the results [5]. Kazimierczak et al. designed their study in such a way that panoramic radiographs and CBCT images were independently evaluated by an orthodontist and a radiologist (both with over 8 years of experience) for the presence of periapical lesions. Each specialist performed their assessment individually in a dimly lit reporting room to ensure optimal conditions for accurate radiological analysis [6]. Szabó et al., in their analysis, evaluated the interproximal surfaces of 323 teeth. These surfaces were assessed independently by two observers as well as the AI system Diagnocat (DGNCT LLC, Miami, FL, USA) for the detection of carious lesions. Four months later, the observers re-evaluated the same images with the assistance of Diagnocat (DGNCT LLC, Miami, FL, USA), and the data were subsequently analyzed using an advanced convolutional neural network (CNN). In the final stage, results with discrepancies between the observers were excluded to estimate the reliability of the system precisely. Despite employing a dual-analysis methodology, the authors concluded that the primary limitation of the study was the absence of clinical validation [11]. Orhan et al., in their study evaluating the accuracy and effectiveness of the Diagnocat (DGNCT LLC, Miami, FL, USA) in identifying dental conditions, analyzed 100 panoramic radiographs (comprising 4497 teeth) sourced from a medical university database. Three radiologists and the Diagnocat (DGNCT LLC, Miami, FL, USA) system independently assessed various dental conditions and treatments, including caries, fillings, calculus deposits, loss of interproximal contact, and periapical lesions. The results were then compared in terms of agreement and sensitivity [7]. The authors emphasize that the lowest reliability in their study was observed in the assessment of caries, periapical lesions, voids in root canals, and overhangs. They also highlight that diagnosing caries is a clinical process requiring the evaluation of disease signs both through direct examination and radiographic imaging.

In the available literature, we found no comparative study conducted as a blind trial where the clinician was unaware that their diagnosis and report would be verified. Another critical factor is the dentist’s age and clinical experience, including training, internships, and specializations. Most dentists are general practitioners without specialization (according to data from Poland [12]), making it reasonable to compare their diagnoses with those of AI systems. Furthermore, modern technologies, including AI-assisted decision-support systems, are typically adopted more quickly by younger dentists, who are accustomed to using technology in their daily lives beyond work.

It is crucial for new technologies to assist clinicians, regardless of their experience level, in detecting diseases at the earliest stages, particularly when symptoms are not yet visible to the naked eye. Combining artificial intelligence with clinical expertise can enhance diagnostic accuracy and improve the effectiveness of early caries detection, ultimately benefiting patients’ health [9,13].

Caries is one of the most common diseases affecting teeth, and its early detection is critical for oral and overall health [14]. Caries develop gradually, with their initial stages often asymptomatic. Early diagnosis facilitates timely and cost-effective treatment decisions, improving patient outcomes [15]. Early detection of caries allows for the use of less invasive treatment methods, such as remineralization, which can halt the progression of the disease without the need for endodontic intervention or, in extreme cases, surgical procedures [16].

Moreover, early treatment of caries prevents complications such as pulpitis or infections, which can lead to more complex health issues and higher treatment costs. Regular check-ups, supported by modern diagnostic technologies, enable the early detection of caries, which is essential for maintaining oral health, improving patient comfort, and reducing the long-term costs of dental care [17,18].

Caries diagnostics rely on several methods that enable early and precise detection of carious lesions. The traditional approach involves clinical examination, during which a dentist assesses the condition of the patient’s teeth using a mirror and probe to identify visible carious changes. This clinical assessment is often complemented by radiographic imaging, which allows for the detection of interproximal and subsurface caries that are not visible to the naked eye.

Modern diagnostic technologies, such as laser fluorescence (e.g., DIAGNOdent devices) and devices utilizing near-infrared (NIR) radiation, offer advanced capabilities for caries detection. Laser fluorescence technology measures light emission from teeth, enabling the detection of caries at very early stages. On the other hand, NIR imaging utilizes near-infrared radiation to identify structural changes in enamel, providing a non-invasive and highly sensitive method for detecting early carious lesions [19].

Additionally, artificial intelligence (AI)-based systems are being developed to analyze diagnostic images and automatically identify carious lesions. The growing interest in AI technology in medicine and dentistry stems from its potential to enhance diagnostic precision, improve treatment efficiency, and reduce healthcare costs [20].

The growing significance of artificial intelligence in medicine, particularly in dentistry, highlights the need for its application in the diagnosis of complex cases, such as caries detection, based on large volumes of diagnostic data. The lack of studies considering real-world clinical conditions, along with the necessity to compare the effectiveness of AI systems with that of general practitioners, justifies the need for this analysis.

Therefore, this study aimed to assess the effectiveness of the AI-based system Diagnocat (DGNCT LLC, Miami, FL, USA) in detecting primary and secondary carious lesions compared to clinical evaluations conducted by three general dentists.

## 2. Material and Method

### 2.1. Description of the Study Population

This was a cross-sectional study involving 127 patients who presented for comprehensive clinical evaluations at Kwiatek Dental Clinic (Poznań, Poland). The study population included 59 men and 68 women aged 18 to 68 years. The clinical evaluations were performed by three dentists of the same gender, each with comparable clinical experience of at least 5 years. Patients were consecutively enrolled during the study period as they presented to the clinic and met the inclusion criteria without a pre-determined sample size.

A review of original studies cited in our bibliography on AI applications in dentistry indicates that they analyzed fewer teeth and/or patients than the present study [1,5,6,7,8,9,11,21,22,23,24,25,26]. Many of these studies focused on AI performance using smaller datasets, either by evaluating a limited number of teeth per patient or relying solely on image-based assessments. In contrast, our study included 3622 analyzed teeth from 127 patients, making it one of the more extensive datasets in AI research applied to dentistry. The scale of our dataset provides a solid foundation for statistical analysis and enhances its relevance to real-world clinical applications.

The inclusion criteria were:

- patients who presented for a comprehensive clinical evaluation at Kwiatek Dental Clinic during the study period,
- age ≥ 18 years,
- availability of a panoramic radiograph.

The exclusion criteria were:

- age < 18 years,
- pregnancy,
- absence of a panoramic radiograph,
- edentulism.

### 2.2. Research Procedure

#### 2.2.1. Description of the AI Software (Diagnocat, DGNCT LLC, Miami, FL, USA)

**Figure 1:**
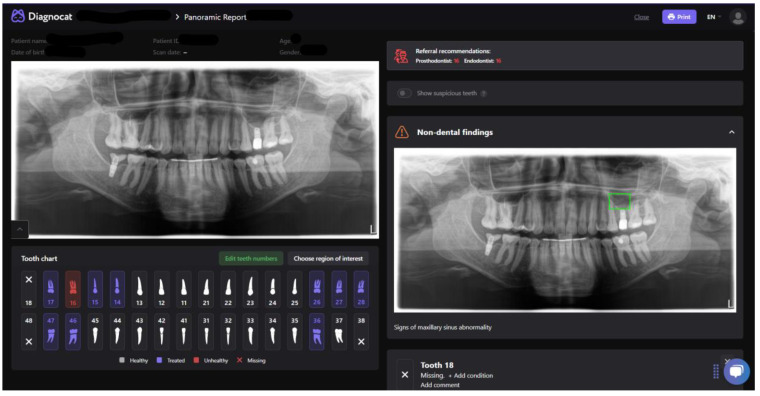
Screenshot of the AI Program “Diagnocat” (DGNCT LLC, Miami, FL, USA)—Report from Panoramic Radiograph Analysis.

Diagnocat (DGNCT LLC, Miami, FL, USA) is an artificial intelligence-based software designed to support imaging diagnostics in dentistry (Figure 1). The system employs machine learning technology to analyze radiographic images, cone-beam computed tomography (CBCT), and other types of dental imaging. It enables the automated identification and classification of various pathologies, such as caries, inflammatory changes, and structural anomalies. The system is regularly updated using new data, including input from clinicians, allowing for continuous improvement in its algorithms [6,21].

#### 2.2.2. Data Collection Process

The study was cross-sectional and prospective. Patients presenting for a comprehensive clinical evaluation during the study period were randomly assigned to a specific dentist. The dentist ordered a panoramic radiographic examination using the Orthophos SL 3D device (Dentsply Sirona, Charlotte, NC, USA). Based on the clinical examination and the obtained radiograph, the dentist diagnosed the presence of caries—either primary or secondary lesions. The same radiograph was then analyzed using the Diagnocat (DGNCT LLC, Miami, FL, USA).

To ensure the objectivity of the study, none of the dentists were informed about the dental diagram analysis or the ongoing comparative study. This approach was designed to directly replicate the clinical scenario in which consultations and diagnoses occur under real-world conditions. The dentists also did not have access to the Diagnocat (DGNCT LLC, Miami, FL, USA) results during their analysis.

To minimize errors associated with early career experience, the study included three dentists, each with a minimum of five years of professional practice.

#### 2.2.3. Statistical Analysis Methods

A comparative analysis was performed to evaluate the detection of caries in the form of primary and secondary lesions by the AI software and the concordance of these results with the clinical assessment conducted by the dentist.

Statistical analyses were performed using Statistica software version 13. Descriptive statistics included means, standard deviations (SD), medians, interquartile ranges, and 95% confidence intervals (CI).

To compare results and evaluate the concordance between Diagnocat (DGNCT LLC, Miami, FL, USA) analyses and clinical evaluations, the Chi-squared test (Chi^2^) was employed. This test allowed for the comparison of proportions across different groups, such as patient gender, age, and types of analyzed teeth. Additionally, the test facilitated the identification of differences in Diagnocat’s (DGNCT LLC, Miami, FL, USA) assessments depending on the dentists conducting the evaluations.

Comparative analyses between groups also utilized the percentage structure index test, providing a detailed evaluation of statistical differences.

To assess the agreement between Diagnocat (DGNCT LLC, Miami, FL, USA) and clinical evaluations across different types of teeth and age groups, an analysis of variance (ANOVA) was performed. Statistical significance tests further expanded the results, identifying significant differences between groups.

Statistical significance was determined based on a *p*-value, with *p* < 0.05 considered statistically significant. Key results of the analysis are presented in graphs and tables, with *p*-values < 0.05 highlighted in bold. Results close to statistical significance (0.05 ≤ *p* < 0.1) were underlined to indicate potential trends warranting further investigation.

### 2.3. Evaluation Criteria

#### 2.3.1. Definition of Primary and Secondary Carious Lesions

Caries are caused by the action of acids produced by cariogenic (caries-forming) bacteria residing on the tooth surface [27].

A primary carious lesion is defined as the loss of hard dental tissues in an area that has not previously undergone treatment or filling. A secondary carious lesion is defined as the loss of hard dental tissues that occurs around or beneath an existing filling, resulting from filling leakage, insufficient removal of caries during previous treatment, or inadequate oral hygiene, leading to the recurrence of the carious process [28]. These definitions align with the guidelines presented in Oral Health Surveys: Basic Methods, 5th Edition, published by the World Health Organization (WHO), ensuring consistency with internationally recognized standards [29]. This means that the existing filling must be removed, the tooth re-prepared, and a new filling placed. Teeth without the changes described above were simply referred to as healthy teeth.

#### 2.3.2. Categories of Analysis: Gender, Age, Dentist

The overall detectability of primary and secondary carious lesions was examined, with a separate statistical analysis verifying the detectability of primary lesions alone.

The analysis included detectability for each of the 32 teeth potentially present in the oral cavity, excluding missing teeth for individual patients. Potential differences were analyzed concerning patient gender, age, tooth groups (incisors, canines, premolars, and molars), specific teeth (11–48), and the dentist conducting the evaluation.

The dentists whose diagnoses were utilized in this study classified teeth for treatment based on panoramic radiographs as well as their clinical experience and medical knowledge. The study was conducted under single-blind conditions, meaning that the dentists were unaware that their evaluations would be subject to detailed scientific analysis.

The inclusion of tooth groups in the analysis was motivated by practical considerations—the differences in the radiological evaluation of these teeth due to the specifics of their anatomy [30].

The analysis accounted for gender differences, as women may experience variations in bone density due to hormonal changes, particularly during menopause, which can impact oral health and the accuracy of radiological evaluation [31]. Men, on the other hand, may have a different bone and dental structure compared to women, which could influence radiological results.

The age-based division was also motivated by potential diagnostic differences: in younger patients, radiological diagnostic challenges may arise due to incomplete root development and tooth crowding [32]. In older individuals, diagnostic challenges may be associated with an increased risk of root caries, the presence of periodontal disease, and complex structural changes [33].

## 3. Results

**Figure 2:**
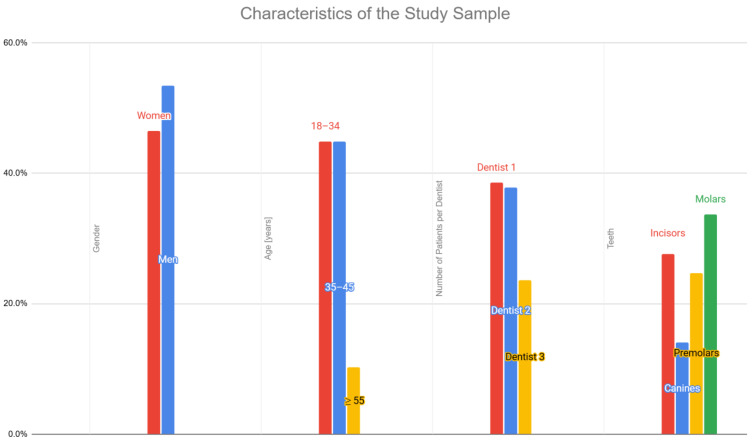
Characteristics of the Study Sample.

The demographic and clinical characteristics of the study population are presented in Table 1 and Figure 2, providing an overview of the patients included in the analysis.

### 3.1. Overall Effectiveness of Diagnocat (DGNCT LLC, Miami, FL, USA)

The reference point in this analysis was the diagnosis established through clinical evaluation. A total of 3622 teeth were examined among the 127 patients, as 442 teeth were missing (including third molars, commonly known as “wisdom teeth”).

Diagnocat (DGNCT LLC, Miami, FL, USA) detected 302 carious lesions (primary or secondary), whereas the dentists identified 834 carious lesions (primary or secondary). The software incorrectly indicated the presence of primary or secondary caries in 136 teeth and failed to detect ongoing carious processes in 668 teeth (Table 2).

### 3.2. Analysis of the Total Number of Primary and Secondary Carious Lesions

**Figure 3:**
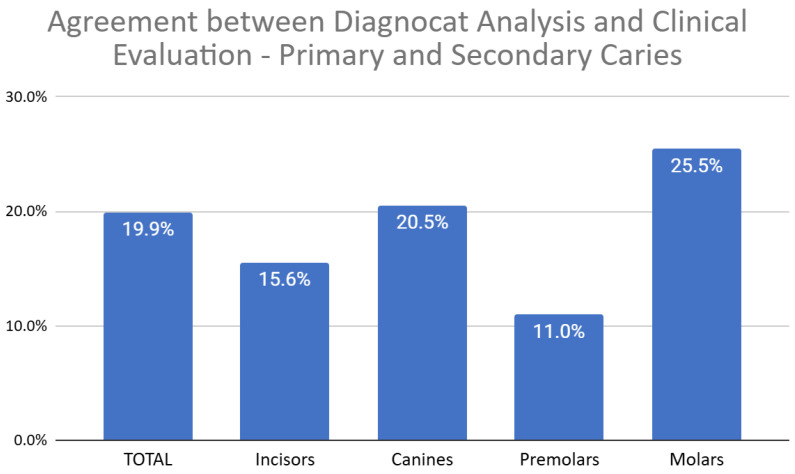
Agreement Between Diagnocat (DGNCT LLC, Miami, FL, USA) Analysis and Clinical Evaluation—Primary and Secondary Caries.

This analysis considered both primary and secondary carious lesions combined. The average agreement between Diagnocat (DGNCT LLC, Miami, FL, USA) analysis and the clinical-radiological assessment by dentists was 19.9%. The lowest agreement rate was observed in premolars (11%), while the highest agreement rate was noted in molars (25.5%) (Figure 3).

#### 3.2.1. Agreement Between Diagnocat (DGNCT LLC, Miami, FL, USA) Analysis and Clinical Evaluation by Gender

**Figure 4:**
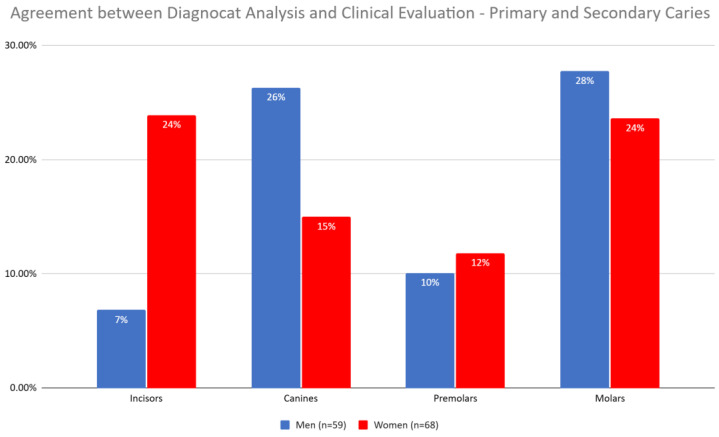
Agreement Between Diagnocat (DGNCT LLC, Miami, FL, USA) Analysis and Clinical Evaluation—Primary and Secondary Caries in Women and Men.

Differences in the agreement between Diagnocat (DGNCT LLC, Miami, FL, USA) analysis and the dental diagnosis were observed based on gender (Table 3, Figure 4).

Comparing the agreement between Diagnocat (DGNCT LLC, Miami, FL, USA) analysis and the clinical evaluation, significant differences in the detection of primary and secondary caries were noted for incisors (*p* = 0.0261, V-square = 4.95), with significantly higher agreement for Diagnocat (DGNCT LLC, Miami, FL, USA) in women (23.9%) compared to men (6.8%) (Table 4).

**Figure 5:**
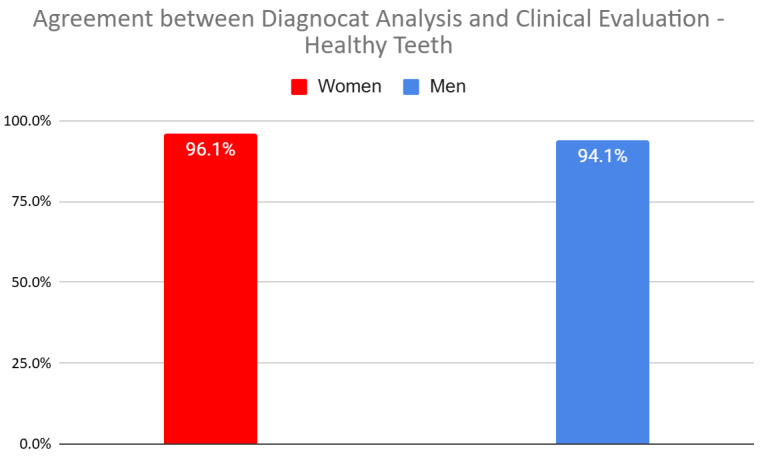
Agreement Between Diagnocat (DGNCT LLC, Miami, FL, USA) Analysis and Clinical Evaluation—Healthy Teeth in Women and Men.

The statistical analysis revealed significant differences between women and men, particularly regarding the agreement between Diagnocat (DGNCT LLC, Miami, FL, USA) analysis and clinical evaluation in detecting the absence of caries (healthy teeth) for all teeth considered collectively (Figure 5). The software diagnosed healthy teeth in women significantly better than men (*p* = 0.014). When analyzing individual tooth groups, results close to statistical significance were observed for premolars and molars (Table 5).

#### 3.2.2. Agreement Between Diagnocat (DGNCT LLC, Miami, Fl, USA) Analysis and Clinical Evaluation by Patient Age

The results were analyzed in the following age categories: 18–34 years (57 subjects), 35–54 years (57 subjects), and 55 and older (13 subjects).

This division into groups was based on differences in caries risk, biological and physiological changes, and varying levels of oral health prevention and hygiene in these age groups.

This classification has already been used in the literature concerning the monitoring of oral health in the population, taking into account specific differences in biology, physiology, and prevention across different age groups [34,35].

**Figure 6:**
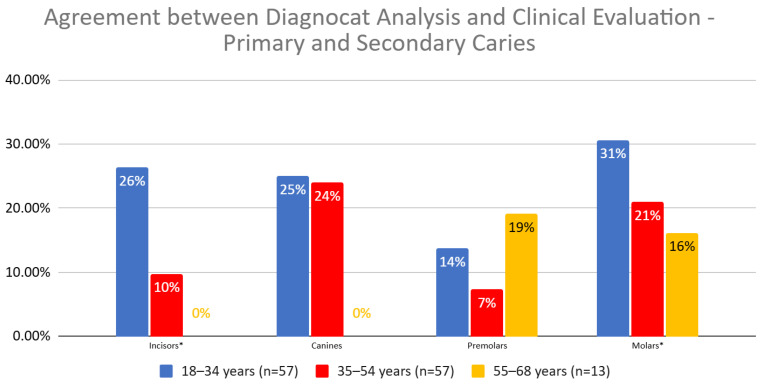
Agreement Between Diagnocat (DGNCT LLC, Miami, FL, USA) Analysis and Clinical Evaluation in Detecting Primary and Secondary Caries by Age Group. (*—Statistically significant difference detected for this tooth type).

The joint statistical analysis of all teeth revealed significant differences in the agreement between Diagnocat (DGNCT LLC, Miami, FL, USA) and clinical evaluation for the different age groups (Pearson Chi-squared = 14.07, *p* = 0.0009). The percentage structure index test showed that the differences were significant between the youngest patient group (18–34 years) and both of the older age groups (35–54 and 55+ years). Greater agreement was observed in the youngest patients (Figure 6, Table 6).

The agreement between Diagnocat (DGNCT LLC, Miami, FL, USA) analysis and clinical evaluation revealed significant differences depending on the patients’ age and the type of teeth assessed. The overall agreement was highest in the 18–34 years group (25.53%), gradually decreased in the 35–54 years group (15.58%), and was lowest in the 55–68 years group (13.04%). The Chi-squared test showed the significance of these differences (*p* = 0.0009). In the percentage structure index test, statistically significant differences were noted between the youngest group and the middle group (*p* = 0.0007), as well as between the youngest and oldest groups (*p* = 0.0246).

For incisors, Diagnocat (DGNCT LLC, Miami, FL, USA)’s agreement was significantly higher in the 18–34 years group (26.32%) compared to the 35–54 years (9.76%) and 55–68 years groups (0%). This difference reached statistical significance (*p* = 0.0044), and the trend was confirmed in the percentage structure index test between groups 1 vs. 2 (*p* = 0.0541) and 1 vs. 3 (*p* = 0.0656), indicating a close significance.

For canines, the highest agreement percentage was noted in the 18–34 years group (25%), but in the older groups, the result dropped to 20% in the middle group and 0% in the oldest group. The differences were not statistically significant (*p* = 0.3080).

Analysis of premolars showed a tendency for higher agreement in the oldest group (15.38%) compared to the middle group (7.32%), although this difference did not reach statistical significance (*p* = 0.3808). However, the percentage structure index test for the comparison between groups 2 vs. 3 (*p* = 0.0803) was close to statistical significance.

For molars, the agreement was highest in the 18–34 years group (30.60%), 20.96% in the middle group, and decreased to 15.09% in the oldest group. These differences did not reach statistical significance (*p* = 0.3018), although the comparison between groups 1 vs. 3 (*p* = 0.0932) indicated a trend approaching significance.

In summary, significantly higher agreement between Diagnocat (DGNCT LLC, Miami, FL, USA) and clinical evaluation was observed in younger patients, particularly in the assessment of incisors. For premolars, there was a tendency for greater agreement in the oldest group compared to the middle group, although these differences were not statistically significant (Table 7).

The agreement between Diagnocat (DGNCT LLC, Miami, FL, USA) analysis and clinical evaluation was also analyzed for individual teeth across different age groups. Significant differences were observed for tooth 36 (*p* = 0.0335) and tooth 44 (*p* = 0.0187), where the agreement was higher in the oldest age group (55–68 years) compared to the other groups (Table 8). In the analysis of agreement for individual teeth, results close to statistical significance were also observed. For tooth 12, higher agreement was noted in the 18–34 years group (66.7%), while in the 35–54 years group, it was 22.2%, and no agreement was found in the 55–68 years group (0%). For tooth 28, the highest agreement was observed in the 35–54 years group (66.7%), while in the 18–34 years and 55–68 years groups, the agreement was 0%.

#### 3.2.3. Agreement Between Diagnocat (DGNCT LLC, Miami, FL, USA) Analysis and Clinical Evaluation by Dentist

**Figure 7:**
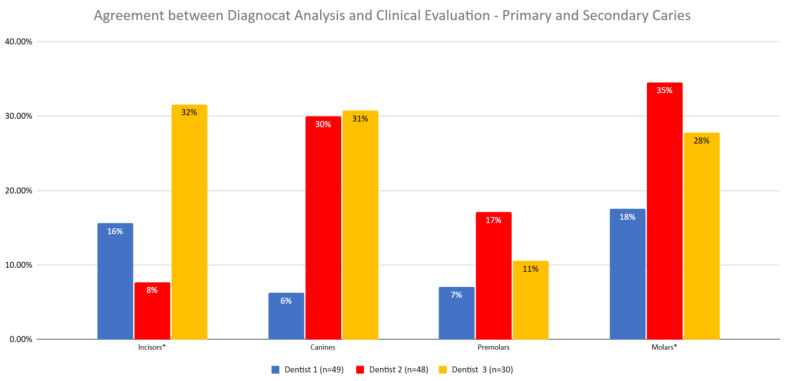
Agreement Between Diagnocat (DGNCT LLC, Miami, FL, USA) Analysis and Clinical Evaluation by Dentist—Primary and Secondary Caries. (*—Statistically significant difference detected for this tooth type).

The agreement between Diagnocat (DGNCT LLC, Miami, FL, USA) analysis and clinical evaluation was examined to determine if it varied depending on the dentist performing the evaluation (Figure 7, Table 9).

The Pearson Chi-squared test result of 16 and a *p*-value of 0.0003 indicate that these differences were statistically significant. The diagnosis of Dentist 1 differed from those of Dentists 2 and 3.

After further analysis of individual tooth groups, it was found that for molars, the agreement between the dentist and Diagnocat (DGNCT LLC, Miami, FL, USA) was significantly lower for Dentist 1 compared to Dentists 2 and 3, and for premolars, the agreement was lower for Dentist 1 compared to Dentist 2.

For Dentists 2 and 3, significantly different agreement levels with Diagnocat (DGNCT LLC, Miami, FL, USA) were observed in the assessment of incisors, with a higher agreement for Dentist 3 (Table 10).

An analysis of the agreement between the evaluations of individual teeth was also conducted, taking into account the results of three dentists. The analysis of the agreement between the dentists for specific teeth revealed significant differences, particularly for teeth 13, 27, and 48.

Tooth 13: The greatest discrepancies were observed between Dentist 1 (0%) and Dentist 3 (67%). Dentist 2 achieved an intermediate result (20%), indicating a lack of consistency in the evaluation between the examiners. Tooth 27: High agreement was observed in Dentist 2 (50%), while Dentist 1 and Dentist 3 achieved 9% and 25%, respectively, showing significant differences in the evaluation between the examiners. Tooth 48: The highest agreement was observed in Dentist 2 (75%), while both Dentist 1 and Dentist 3 rated the agreement at 0%. For teeth 24 and 25, the *p*-values were close to the threshold of statistical significance, suggesting smaller yet noticeable differences in the evaluations. In these cases, the differences were primarily between Dentist 2 and the other dentists. These results highlight the lack of full diagnostic consistency between the examiners, particularly for selected teeth where discrepancies were most apparent (Table 11).

### 3.3. Analysis of Primary Caries Detection

**Figure 8:**
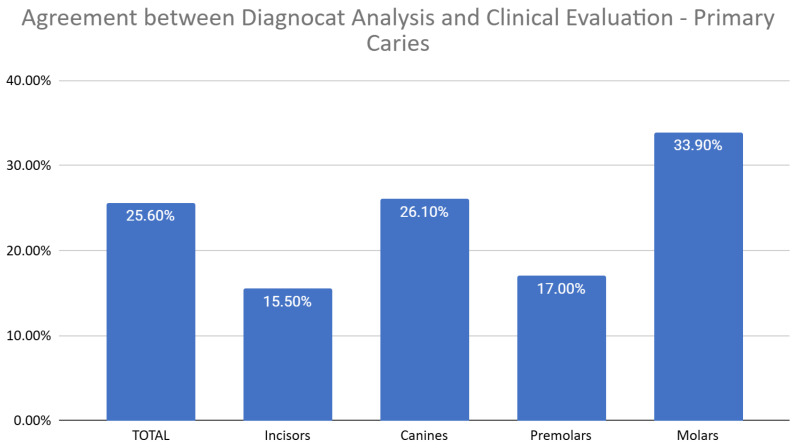
Agreement Between Diagnocat (DGNCT LLC, Miami, FL, USA) Analysis and Clinical Evaluation—Primary Carie.

This analysis focused solely on evaluating the agreement between Diagnocat (DGNCT LLC, Miami, FL, USA) and clinical evaluation concerning primary carious lesions, i.e., carious changes, without including factors such as faulty fillings. The analysis revealed differences in the level of agreement depending on the type of teeth, with the highest agreement observed for molars (33.9%) and the lowest for incisors (15.5%). Detailed results for individual tooth groups are presented in the following chart (Figure 8).

#### 3.3.1. Agreement Between Diagnocat (DGNCT LLC, Miami, FL, USA) Analysis and Clinical Evaluation by Gender

**Figure 9:**
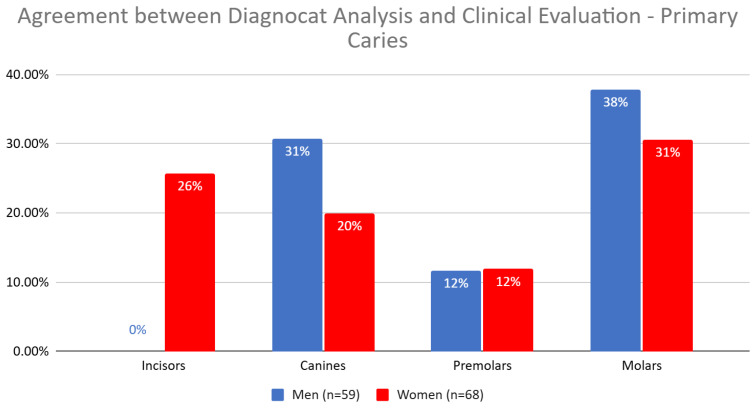
Agreement Between Diagnocat (DGNCT LLC, Miami, FL, USA) Analysis and Clinical Evaluation—Primary Caries in Women and Men.

The analysis of the agreement between Diagnocat (DGNCT LLC, Miami, FL, USA) and clinical evaluation revealed varying detection rates of primary caries depending on gender and tooth type. For men, caries were most frequently detected in molars, while for women, caries were detected in both incisors and molars. For canines and premolars, the detection rate was similar between the groups, with a slight advantage for men in the case of canines (Table 12, Figure 9).

When comparing the agreement between Diagnocat (DGNCT LLC, Miami, FL, USA) analysis and clinical evaluation, significant differences in the detection of primary caries for incisors were observed depending on the patients’ gender (*p* = 0.0229). The agreement was clearly higher in women (25.71%) compared to men (0.00%), which was also confirmed by the percentage structure index test (*p* = 0.0082) (Table 13).

#### 3.3.2. Agreement Between Diagnocat (DGNCT LLC, Miami, USA) Analysis and Clinical Evaluation by Patient Age

**Figure 10:**
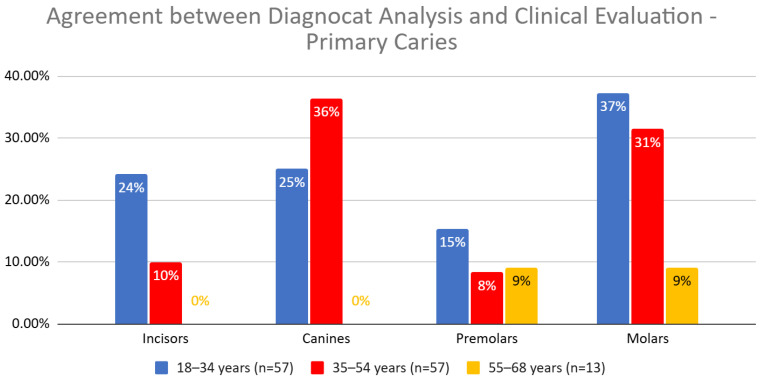
Agreement Between Diagnocat (DGNCT LLC, Miami, FL, USA) Analysis and Clinical Evaluation in Detecting Primary Caries by Age Group.

The agreement between Diagnocat (DGNCT LLC, Miami, FL, USA) analysis and clinical evaluation for primary caries varied depending on the age group. In the 18–34 years group, the highest agreement was observed for molars (37%), followed by canines (25%) and incisors (24%), while premolars reached a result of 15%. In the 35–54 years group, the highest agreement was for canines (36%) and molars (31%), with lower agreement for incisors (10%) and premolars (8%). In the 55–68 years group, the agreement was the lowest, with results at 9% for premolars and molars and 0% for incisors and canines (Table 14, Figure 10).

The agreement between Diagnocat (DGNCT LLC, Miami, FL, USA) analysis and clinical evaluation revealed significant differences between age groups (*p* = 0.0045). The highest agreement was observed in the 18–34 years group, with an agreement rate of 28.85%, while in the 35–54 years group, it dropped to 20.83%, and in the oldest group (55–68 years), it was only 5.71%.

Analysis of individual tooth types indicated that for incisors, the highest agreement was found in the 18–34 years group (24.14%), while in the middle-aged group, the agreement was 10%, and in the oldest group, no agreement was observed (0%). The result for incisors was not statistically significant (*p* = 0.0819), although the difference between the youngest and oldest groups (*p* = 0.1027) was close to statistical significance.

For canines, the highest agreement rate was observed in the 35–54 years group (36.36%), with 25% in the 18–34 years group and no agreement in the oldest group (0%). These differences were not statistically significant (*p* = 0.2249).

For premolars, the agreement was similar in the youngest and oldest groups, at 15.38% and 9.09%, respectively, while in the middle group, it decreased to 8.33%. Despite the lack of statistical significance (*p* = 0.3891), the difference between the 18–34 years and 35–54 years groups (*p* = 0.1843) was close to statistical significance.

For molars, the highest agreement was observed in the 18–34 years group (37.24%), with the middle group showing 31.46%. The lowest agreement was found in the oldest group, at 9.09%. These differences were not statistically significant (*p* = 0.0957), although the comparison between the 18–34 years and 55–68 years groups (*p* = 0.0596) was close to statistical significance.

In summary, the overall agreement between Diagnocat (DGNCT LLC, Miami, FL, USA) and clinical evaluation was highest in the youngest group and systematically decreased with age, with the differences between the youngest and oldest groups being particularly noticeable for incisors and molars (Table 15).

The agreement between Diagnocat (DGNCT LLC, Miami, FL, USA) analysis and clinical evaluation for individual teeth across different age groups was also analyzed. For tooth 13, a result close to statistical significance was observed (*p* = 0.0880), with the highest agreement observed in the 35–54 years group (57.1%), while in the other age groups, the agreement was 0% (Table 16).

#### 3.3.3. Agreement Between Diagnocat (DGNCT LLC, Miami, FL, USA) Analysis and Clinical Evaluation by Dentist

**Figure 11:**
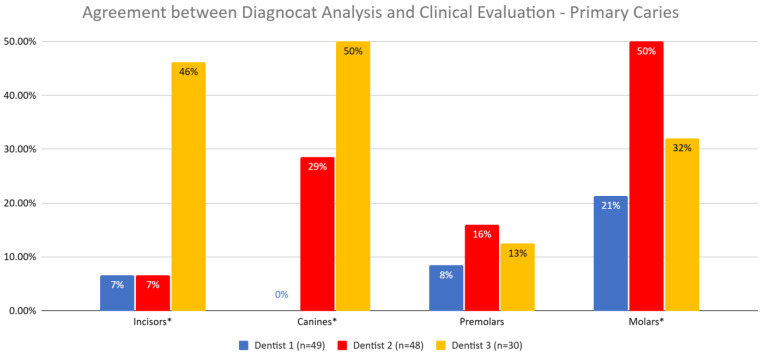
Agreement Between Diagnocat (DGNCT LLC, Miami, FL, USA) Analysis and Clinical Evaluation by Dentist—Primary Caries. (*—Statistically significant difference detected for this tooth type).

The agreement between Diagnocat (DGNCT LLC, Miami, FL, USA) analysis and clinical evaluation varied depending on the dentist and tooth type. For incisors, the highest agreement was observed with Dentist 3 (46%), while Dentist 1 and Dentist 2 achieved the same result of 7%. For canines, the highest agreement was again observed with Dentist 3 (50%), while Dentist 2 achieved 29%, and Dentist 1 found no agreement (0%). For premolars, the values were lower, with the highest result for Dentist 2 (16%) and lower values for Dentist 1 (8%) and Dentist 3 (13%). For molars, the highest agreement was observed with Dentist 2 (50%), while Dentist 3 achieved 32%, and Dentist 1 reached 21% (Table 17, Figure 11).

The agreement between Diagnocat (DGNCT LLC, Miami, FL, USA) analysis and clinical evaluation differed significantly depending on the dentist (*p* = 0.0004). The overall agreement was highest with Dentist 2 (30.91%) and Dentist 3 (28.78%), while the lowest value was observed with Dentist 1 (14.21%). Statistically significant differences were observed between Dentist 1 and Dentist 2 (*p* = 0.0002) as well as between Dentist 1 and Dentist 3 (*p* = 0.0013).

For incisors, the agreement was significantly higher with Dentist 3 (46.15%) compared to Dentists 1 and 2, who achieved identical results (6.67%). These differences were statistically significant between Dentist 1 and Dentist 3 (*p* = 0.0161) as well as between Dentist 2 and Dentist 3 (*p* = 0.0022).

For canines, the highest agreement was observed with Dentist 3 (50%), followed by Dentist 2 (28.57%), while Dentist 1 found no agreement (0%). The difference between Dentist 1 and Dentist 3 was statistically significant (*p* = 0.0209).

For premolars, the values were lower, and the differences between dentists were not statistically significant (*p* = 0.4424), with the highest agreement observed with Dentist 2 (16.00%) and slightly lower values with Dentist 1 (8.45%) and Dentist 3 (12.50%).

For molars, the highest agreement was observed with Dentist 2 (50%), while Dentist 3 achieved 32.05%, and Dentist 1 reached 21.35%. Statistically significant differences were observed between Dentist 1 and Dentist 2 (*p* = 0.0001) as well as between Dentist 2 and Dentist 3 (*p* = 0.0227).

In summary, the agreement between Diagnocat (DGNCT LLC, Miami, FL, USA) analysis and clinical evaluation was highest with Dentist 2 and Dentist 3, particularly in the assessment of molars and incisors. Statistically significant differences were mainly observed in comparisons with Dentist 1, suggesting variability in the evaluation of different tooth types between the dentists (Table 18).

The analysis of the agreement for primary caries revealed significant differences for teeth 13 (*p* = 0.0090), 27 (*p* = 0.0275), and 48 (*p* = 0.0085). The greatest discrepancies were observed between the dentists, particularly for tooth 48, where agreement occurred only with Dentist 2 (100%), while the other dentists achieved 0%. For tooth 13, the highest agreement was observed with Dentist 3 (80%), while for tooth 27, the values were more varied between the examiners (Table 19).

### 3.4. Analysis of the Impact of Patient Group Structure (Age, Gender) on the Agreement Between Diagnocat (DGNCT LLC, Miami, FL, USA) and Doctors’ Evaluations

To accurately interpret the conclusions drawn above, additional statistical analyses were performed, providing detailed information on the relationships between the variables (gender, age, dentist) and allowing for the description of statistical differences and associations between the studied groups. The results indicated statistical significance for some relationships, which could be important for data interpretation and inference in the context of the study.

The data showed a similar age distribution between women and men, with some differences in variability but no statistically significant differences. Statistically significant differences were found in the gender distribution between doctors (*p* < 0.05). Women were more often patients of Doctors 1 and 2, while men were more frequently patients of Doctor 3 (Table 20).

## 4. Discussion

### 4.1. Overall Effectiveness of Diagnocat (DGNCT LLC, Miami, FL, USA) Compared to Clinical Evaluations

**Figure 12:**
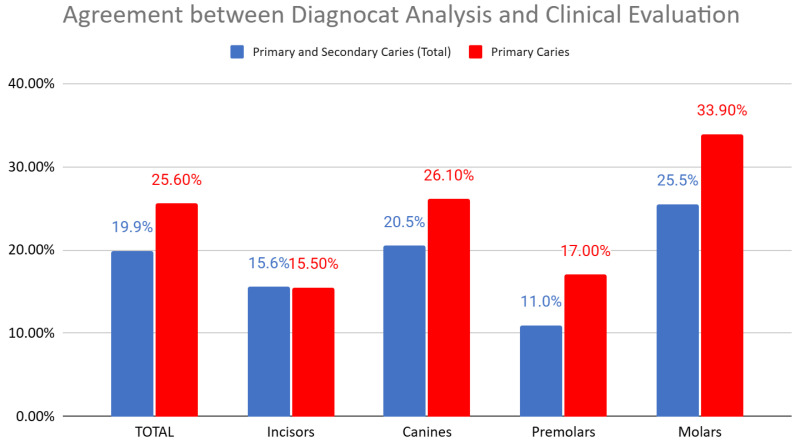
Agreement Between Diagnocat (DGNCT LLC, Miami, FL, USA) Analysis and Clinical Evaluation—Primary and Secondary Caries (Total) and Primary Caries.

This study assessed the overall effectiveness of the Diagnocat (DGNCT LLC, Miami, FL, USA) system in caries diagnosis, comparing its performance with that of three dentists. The analysis revealed variability in agreement between Diagnocat (DGNCT LLC, Miami, FL, USA) and clinical evaluations, depending on the type of caries. The system demonstrated higher accuracy in detecting primary caries compared to the combined detection of primary and secondary caries, suggesting that identifying caries in untreated teeth is more straightforward for the AI system (Figure 12). This aligns with previous research indicating that secondary caries diagnosis presents a significant clinical challenge [36].

### 4.2. Differences in Detection Effectiveness Based on Patient Gender

The analysis revealed differences in the agreement between Diagnocat’s (DGNCT LLC, Miami, FL, USA) results and clinical evaluations depending on patient gender. For incisors, significantly higher agreement was observed in women compared to men, both for the total number of primary and secondary caries and for primary caries alone.

The good sensitivity in the assessment of healthy teeth in the conducted study is consistent with the findings of other researchers [7,37]. The agreement between Diagnocat (DGNCT LLC, Miami, FL, USA) and clinical evaluation differed statistically between women and men. Significantly higher detection of healthy teeth (across all groups) was observed in women, along with a tendency for better agreement between Diagnocat (DGNCT LLC, Miami, FL, USA) and clinical evaluation in women, particularly in the assessment of premolars and molars.

Based on the knowledge that anatomical differences between teeth may complicate the diagnosis of caries on panoramic radiographs [9], and the density and structure of enamel may differ slightly between women and men, we can interpret these results as follows: the higher sensitivity of Diagnocat (DGNCT LLC, Miami, FL, USA) in diagnosing caries in incisors in women is likely due to their simpler anatomical structure, better visibility on radiographs due to their position in the anterior part of the dental arch, and fewer radiological artifacts that could interfere with the analysis.

### 4.3. Differences in Detection Effectiveness Based on Patient Age

The agreement between Diagnocat (DGNCT LLC, Miami, FL, USA) results and clinical evaluation significantly varied between age groups, with the highest agreement in the youngest patient group (18–34 years). This may be due to better radiographic visibility resulting from a lower number of structural changes, such as fillings or enamel wear. In older patients (35–54 and 55–68 years), these changes, along with the presence of secondary caries, may have hindered the interpretation of images.

The analysis revealed significant differences in detection for specific teeth. For example, in the case of molars (e.g., tooth 36), the agreement was higher in the oldest patient group (55–68 years), which may be due to more advanced carious changes, making diagnosis easier. In contrast, for incisors (e.g., tooth 12) and selected molars (e.g., tooth 37), the highest effectiveness was observed in the youngest group, which confirms the influence of simpler anatomical structure and better radiographic visibility in this group. Similarly, the study by Zhang et al. confirmed that the diagnostic agreement between the clinician and AI was highest for anterior teeth [37].

These results highlight the importance of both patient age and the anatomical characteristics of individual teeth in the diagnostic effectiveness of Diagnocat (DGNCT LLC, Miami, FL, USA).

### 4.4. Differences in Detection Effectiveness Based on the Dentist Performing the Clinical Evaluation

Dentist 1 showed the lowest agreement with Diagnocat (DGNCT LLC, Miami, FL, USA), particularly in the assessment of incisors and premolars, as seen in both the analysis of primary caries and the total of primary and secondary caries. This may be due to greater diagnostic challenges associated with these tooth groups, such as reduced visibility of interproximal surfaces and the more complex anatomy of premolars. Additionally, the lower agreement could be related to potentially less diagnostic experience of Dentist 1 or a different approach to interpreting radiographic images, which influenced the consistency of results with Diagnocat (DGNCT LLC, Miami, FL, USA).

In contrast to Dentist 1, Dentists 2 and 3 achieved significantly higher agreement with Diagnocat (DGNCT LLC, Miami, FL, USA), which may indicate greater precision in radiographic image analysis and a more developed diagnostic evaluation method. The differences between Dentist 2 and Dentist 3 were statistically significant, mainly for incisors, where Dentist 3 achieved better results. This could be due to a more effective use of experience in identifying subtle carious changes in this group of teeth.

The highest agreement with Diagnocat (DGNCT LLC, Miami, FL, USA) was observed for molars, particularly with Dentist 2. Molars, due to their larger size and better visibility on panoramic radiographs, are easier to assess both for the AI system and for the dentists.

The analysis of Diagnocat (DGNCT LLC, Miami, FL, USA)’s agreement with clinical evaluations for individual teeth confirms the results outlined above, showing the lowest agreement with Dentist 1, particularly for incisors and premolars, and higher agreement with Dentists 2 and 3, especially for molars and selected incisors.

Similar variations in caries diagnosis between dentists were also described in studies by Cantu et al. [22] and Da Silva et al. [38]. In their study, Amsaya et al. demonstrated that the use of the Diagnocat (DGNCT LLC, Miami, FL, USA) system improved both the consistency of assessments among the three observers and their accuracy in diagnosing dental caries on CBCT images [39].

### 4.5. Analysis of Patient Distribution and Its Impact on Results

The results revealed significant differences in the gender distribution of patients between the dentists (*p* = 0.0299), with Dentist 3 having a clear majority of male patients (73.33%), in contrast to Dentist 1 and Dentist 2, whose patients were mostly female. It is important to note that despite previous findings indicating easier detection of caries in women, Dentist 3 achieved the highest agreement with Diagnocat (DGNCT LLC, Miami, FL, USA). However, it should be emphasized that the influence of patient group structure and gender differences on the effectiveness of caries detection requires further investigation. Future analyses should consider not only the gender of patients but also the experience of the dentists and other factors that may influence diagnostic outcomes.

### 4.6. Strengths and Weaknesses of Diagnocat (DGNCT LLC, Miami, FL, USA)

Diagnocat (DGNCT LLC, Miami, FL, USA), as a system based on artificial intelligence and machine learning, has several significant advantages that make it a valuable tool in dental diagnostics. First and foremost is its speed of analysis. Diagnocat (DGNCT LLC, Miami, FL, USA) automates the radiological evaluation process, significantly reducing the time needed to detect carious changes. Unlike traditional analysis performed by a dentist, the system is capable of providing results almost immediately after the radiographic image is uploaded.

Secondly, the automation of the process: the program eliminates some of the subjective errors arising from the interpretation of images by dentists, which increases the repeatability of results and standardizes the evaluation. As a result, it is possible to obtain more consistent analyses, regardless of the diagnostic experience.

It is important to emphasize that Diagnocat (DGNCT LLC, Miami, FL, USA) is not focused on a specific area, which often appears in standard diagnostics. When evaluating radiographic images, dentists may focus on areas that the patient identifies as the source of pain or discomfort. This “focus” can lead to overlooking less obvious changes in other areas. Diagnocat (DGNCT LLC, Miami, FL, USA) allows for the detection of carious changes that might have been missed during traditional evaluation by objectively and systematically analyzing the entire image.

The system can be particularly useful in detecting carious lesions on surfaces that are difficult to assess, as well as identifying changes in the early stages, which may be invisible or overlooked during manual assessment.

Its clinical utility is also significant. Diagnocat (DGNCT LLC, Miami, FL, USA) can serve as a tool to assist dentists in making diagnostic decisions, particularly in situations with limited working time and a high number of patients. This is confirmed by the study by Ezhov et al., who tested the efficiency, speed, and accuracy of Diagnocat (DGNCT LLC, Miami, FL, USA) in detecting pathological changes and anatomical points, concluding that the program can enhance the decision-making processes of clinicians [8].

While Diagnocat (DGNCT LLC, Miami, FL, USA) offers significant clinical benefits, it is essential to recognize that no diagnostic system is without limitations. Certain factors may still affect its diagnostic effectiveness.

First of all, it should be noted that Diagnocat (DGNCT LLC, Miami, FL, USA) in this study analyzed two-dimensional panoramic radiographs (2D), which inherently limit the ability to detect primary and secondary caries on occlusal surfaces. Similar difficulties arise for caries located on the facial and lingual surfaces, which may be obscured by other tooth structures, making their clear identification on 2D images more challenging.

Based on the results obtained and the available literature, it is worth considering the additional acquisition of four bitewing or periapical images after the 2D panoramic radiograph. These images should cover the areas of the premolars, where—as shown by the statistical analysis—the agreement between Diagnocat (DGNCT LLC, Miami, FL, USA) results and clinical evaluations for primary and secondary caries was lowest (11%). One example is premolars, where, due to their position in the dental arch and the overlapping of structures on panoramic radiographs, the risk of errors in Diagnocat’s (DGNCT LLC, Miami, FL, USA) analysis is higher. In these cases, it should be considered to supplement the diagnostic process with bitewing radiographs, which can significantly improve the accuracy of detecting carious changes on interproximal surfaces, as indicated by the literature [23,40,41].

It is important to emphasize that panoramic radiography is primarily used as a screening tool, and its frequency should be tailored to the individual needs of the patient and clinical indications. In contrast, CBCT imaging is limited to situations that require in-depth clinical analysis and treatment planning, such as implantology, surgical, or orthodontic procedures, and its use should be justified according to the “As Low as Reasonably Achievable” principle [30,42].

However, CBCT diagnostics can significantly complement clinical examination and enhance the accuracy of assessments made by artificial intelligence algorithms. An example is the study conducted by Kazimierczak et al. on detecting periapical changes, where the Diagnocat (DGNCT LLC, Miami, FL, USA) system achieved significantly higher sensitivity with CBCT images (77.78%) compared to panoramic radiographs (OPG) (33.33%) [2]. These results show that CBCT offers greater diagnostic accuracy in assessing such changes, which confirms the potential of three-dimensional images in optimizing the performance of AI tools. In many other studies, authors highlight the greater potential of CBCT compared to radiographs—at least in relation to selected tooth surfaces [43,44,45,46,47].

When analyzing the limitations of the Diagnocat (DGNCT LLC, Miami, FL, USA) AI system, it is important to consider its reliance on the quality of the radiographic images provided. Artifacts, low resolution, or technical errors may decrease the precision of the analysis.

Additionally, the AI system operates based on machine learning algorithms that evaluate only radiological features. Diagnocat (DGNCT LLC, Miami, FL, USA) does not take into account the full clinical context of the patient (symptoms, clinical examination), which in some cases may lead to incorrect assessments. The final diagnostic decision should always consider the full clinical picture of the patient as well as the experience of the dentist.

### 4.7. Strengths of the Study

The present study has several key strengths that highlight its significance in the development of AI-assisted diagnostics. First, the use of a single-blind design allowed for the replication of real clinical conditions, in which dentists diagnose patients without being aware that their assessments will undergo further analysis. This approach enhances the credibility and representativeness of the obtained data in relation to everyday dental practice.

Second, this study fills a gap in the literature as one of the few conducted in real clinical settings using a single-blind methodology. Unlike previous studies evaluating the effectiveness of Diagnocat (DGNCT LLC, Miami, FL, USA) under controlled laboratory conditions (e.g., Szabó et al. [11], Issa et al. [1]), our study examines the system’s performance in dynamic and non-standard clinical scenarios, reflecting the challenges of daily practice. A similar research approach was employed by Zhang et al. [37], who also conducted their study in clinical conditions and subsequently compared their findings with AI-based analysis. However, their study was based on intraoral camera images rather than panoramic radiographs. Moreover, the dentists in their study were aware that their diagnostic results would be compared with AI outcomes, which may have subtly influenced their diagnostic approach.

Mohammad-Rahimi et al. [13], in their systematic review, also emphasized the benefits of testing AI models on data derived from real clinical environments, as this allows for a more reliable assessment of their performance and resilience in practical scenarios.

Finally, the inclusion of diverse patient groups (age, sex) and three independent dentists with comparable experience underscores the generalizability of the findings and their potential relevance for clinical practice. This study makes a novel contribution to the literature by demonstrating how Diagnocat (DGNCT LLC, Miami, FL, USA) performs in real-world conditions, where case variability and the lack of full standardization may significantly impact diagnostic outcomes.

### 4.8. Implications of the Results for Clinical Practice

The results of this study have significant implications for daily clinical practice. Although, as in other reports, the sensitivity of caries detection remains limited and requires further training of the system, the literature suggests its utility in detecting many other changes, such as dental calculus and periodontal bone loss [48] or changes in furcations [7] and periapical lesions [37]. The system allows for quick and objective analysis of radiographic images, which can be particularly useful in situations of high workload and limited time for detailed image evaluation.

Radiographs remain the most commonly used and preferred method for caries diagnosis [24]. Therefore, in clinical practice, the system can serve as support for less experienced dentists, eliminating some of the errors resulting from subjective assessments and providing greater consistency in diagnoses. At the same time, Diagnocat (DGNCT LLC, Miami, FL, USA) systematically analyzes the entire radiographic image, allowing for the detection of changes that might be missed during manual evaluation, particularly in areas that were not the source of complaints reported by the patient.

The user-friendly and clear interface of the program can also provide valuable support in communication between the dentist and the patient.

In conclusion, the study results suggest that Diagnocat (DGNCT LLC, Miami, FL, USA) can serve as a complement to traditional dental diagnostics, improving its efficiency and accuracy while maintaining the clinical practitioner’s vigilance and utilizing the full diagnostic context.

### 4.9. Limitations of the Study

The study has several significant limitations that should be considered when interpreting the results. Diagnocat (DGNCT LLC, Miami, FL, USA) analyzed two-dimensional panoramic radiographs, which inherently have limited diagnostic value for detecting caries on occlusal, facial, and lingual surfaces. The overlapping of tooth structures on panoramic images makes it difficult to accurately identify carious changes in these areas.

The study did not include additional imaging techniques, such as bitewing or periapical images, which could significantly increase the effectiveness of caries detection, particularly on the interproximal surfaces of teeth, as confirmed by the literature [23,37].

Differences in the gender and age of patients assigned to individual dentists may have influenced the agreement results. The heterogeneous structure of the study group, including the predominance of male patients with one dentist, may have partly affected the results obtained. Differences in the agreement between Diagnocat (DGNCT LLC, Miami, FL, USA) and clinical evaluation may be due to the individual diagnostic experience of the dentists and variations in radiographic image interpretation. Diagnocat (DGNCT LLC, Miami, FL, USA) based its analysis solely on radiological features without considering symptoms reported by the patient or the results of clinical examination. This could lead to misdiagnosis in cases where carious changes are radiographically invisible but clinically detectable. Interestingly, AI systems that analyze photographic images are currently in the testing phase [25], which could complement the evaluation of radiographic images. Schwendicke et al. highlights the potential of such solutions in improving the accuracy of dental diagnostics and the efficiency of clinical workflows while also emphasizing the challenges associated with their integration into daily practice [4]. The quality of panoramic radiographs, including the presence of artifacts, improper patient positioning, or differences in equipment settings, may have affected the precision of the analyses performed by Diagnocat (DGNCT LLC, Miami, FL, USA).

A limitation of our study may be the assumption that the dental examination performed by the dentists represents the standard diagnostic reference, while we cannot be certain that all evaluations were completely accurate and free from subjective errors. In this context, it is worth noting the findings of Lee et al. and others [9], which indicate that the Diagnocat (DGNCT LLC, Miami, FL, USA) system achieves high effectiveness in caries diagnosis, highlighting the need for further investigation into its potential. The valuable role of AI in assessing carious lesions is also emphasized by Lin et al. [26]

One of the study’s limitations is the lack of a formal analysis of sensitivity and specificity, which stems from the study design, where clinicians evaluated different panoramic radiographs. This methodology was chosen to replicate real clinical conditions; however, it prevents a precise comparison of the agreement between Diagnocat (DGNCT LLC, Miami, FL, USA) and clinicians in the assessment of the same cases.

### 4.10. Recommendations for Future Research

The results of the study highlight areas that require further improvement.

It would be valuable to conduct comparative studies on the effectiveness of Diagnocat (DGNCT LLC, Miami, FL, USA) in detecting caries on CBCT images compared to OPG. Although CBCT is not recommended as a screening tool, it is essential to evaluate the detectability of caries on 3D CBCT images in relation to Diagnocat (DGNCT LLC, Miami, FL, USA) analysis and clinical assessment by a dentist. Such studies could provide valuable insights into the potential of three-dimensional diagnostics for the further development of AI-based tools.

Further research is recommended to explore how Diagnocat (DGNCT LLC, Miami, FL, USA) can improve its accuracy by learning from more diverse datasets encompassing a wide spectrum of clinical cases.

Furthermore, identifying the tooth groups and patient groups where Diagnocat (DGNCT LLC, Miami, FL, USA)’s effectiveness is lower can serve as a starting point for further development of machine learning algorithms. Verifying and improving these aspects will better tailor the AI tool to the needs of clinicians and make it a more effective diagnostic aid. As indicated by other authors, differences in the sensitivity of AI models may stem from the wide range of clinical manifestations of caries, which, in real-world conditions, are more complex than in the selected datasets used for model development [25,37].

Importantly, systems like Diagnocat (DGNCT LLC, Miami, FL, USA) and other tools based on machine learning and neural networks have the ability to continuously improve their effectiveness as the number of data submissions and evaluations increases [49]. Each additional study submitted by users over time improves the sensitivity and specificity of the system, enabling its further optimization.

It is valuable to conduct studies on the effectiveness of AI in various clinical settings, such as in patients at high risk of caries, in children, or in cases of advanced carious lesions. The evaluation of AI performance should also consider different imaging techniques and their impact on improving diagnostics.

Furthermore, the future of AI-based systems should be viewed within a broader diagnostic context. The integration of digital scans, extraoral images, and laboratory data could enable AI systems to detect pathologies beyond basic dental diagnostics, such as early-stage skin malignancies in the head and neck region or mucosal lesions.

As AI-based diagnostic tools continue to evolve, it is crucial that comparative studies are conducted using the latest versions of the system, incorporating the most up-to-date data and algorithm updates.

## 5. Conclusions

The study demonstrated that the diagnostic accuracy of the Diagnocat (DGNCT LLC, Miami, FL, USA) system in detecting carious lesions varied depending on tooth location, patient age, and sex. The highest agreement with clinicians’ assessments was observed for molars and incisors, while the lowest agreement was noted for premolars. The system exhibited greater accuracy in younger patients and showed higher precision in identifying healthy teeth in female patients.

The technological limitations of Diagnocat (DGNCT LLC, Miami, FL, USA) were highlighted, particularly its challenges in diagnosing carious lesions on occlusal and proximal surfaces, indicating the need for complementary analysis using more detailed radiographic imaging. Nevertheless, Diagnocat (DGNCT LLC, Miami, FL, USA) has the potential to serve as a valuable adjunct in dental diagnostics, aligning with the broader necessity for changes in oral healthcare approaches. Modern dentistry requires reforms not only in diagnostic technologies but also in global health policies, as emphasized in the scientific literature [50,51].

To enable the widespread clinical implementation of such tools, further research is necessary, particularly studies conducted in real-world clinical settings.

## Figures and Tables

**Table 1:** Characteristics of the Study Sample.

Parameter	
	*n* (%)
Gender	
Women	59 (46.5%)
Men	68 (53.5%)
Age [years]	
18–34	57 (44.9%)
35–54	57 (44.9%)
≥55	13 (10.2%)
Number of Patients per Dentist	
Dentist 1	49 (38.6%)
Dentist 2	48 (37.8%)
Dentist 3	30 (23.6%)
Teeth	
Incisors	1001 (27.6%)
Canines	506 (14.0%)
Premolars	896 (24.7%)
Molars	1219 (33.7%)

**Table 2:** Overall Agreement and Errors in Diagnocat (DGNCT LLC, Miami, FL, USA) Analysis Compared to Clinical Evaluation in Assessing Tooth Health.

*n* = 127					
	Agreement between Diagnocat (DGNCT LLC, Miami, FL, USA) and clinical evaluation—Healthy Tooth	Missed Detection of Primary and Secondary Caries by Diagnocat (DGNCT LLC, Miami, FL, USA)	False Detection of Primary and Secondary Caries by Diagnocat (DGNCT LLC, Miami, FL, USA)	Agreement between Diagnocat (DGNCT LLC, Miami, FL, USA) and clinical evaluation—Primary and Secondary Caries	Missing Teeth
Total	2652	668	136	166	442

**Table 3:** Agreement Between Diagnocat (DGNCT LLC, Miami, FL, USA) Analysis and Clinical Evaluation—Primary and Secondary Caries in Women and Men.

	Men (*n* = 59)	Women (*n* = 68)
Incisors *	7%	24%
Canines	26%	15%
Premolars	10%	12%
Molars	28%	24%

*—Statistically significant difference detected for this tooth type.

**Table 4:** Comparison of Agreement Between Diagnocat (DGNCT LLC, Miami, FL, USA) Analysis and Clinical Evaluation in Detecting Primary and Secondary Caries for Incisors, Considering Patient Gender.

Teeth	Men (*n* = 59) 46%	Women (*n* = 68) 54%	Test Result *	*p*-Value	*p*-Value
	Agreement Between Diagnocat (DGNCT LLC, Miami, FL, USA) Analysis and Clinical Evaluation				Percentage Structure Index Test
Incisors	6.8%	23.9%	4.95	0.0261	0.0253

Note: *—V-square.

**Table 5:** Comparison of Agreement Between Diagnocat (DGNCT LLC, Miami, FL, USA) Analysis and Clinical Evaluation for Different Groups of Healthy Teeth in Women and Men.

	Men (*n* = 59)	Women (*n* = 68)	Test Result *	*p*-Value
	Agreement Between Diagnocat (DGNCT LLC, Miami, FL, USA) Analysis and Clinical Evaluation—Healthy Teeth			
Total	94.1%	96.1%	5.95	0.0147
Premolars	94.0%	96.7%	2.79	0.0952
Molars	87.0%	91.0%	3.02	0.0821

Note: *—Pearson Chi-squared.

**Table 6:** Agreement Between Diagnocat (DGNCT LLC, Miami, FL, USA) Analysis and Clinical Evaluation by Tooth Type and Patient Age Group—Primary and Secondary Caries.

	18–34 Years (*n* = 57)	35–54 Years (*n* = 57)	55–68 Years (*n* = 13)
Incisors *	26%	10%	0%
Canines	25%	24%	0%
Premolars	14%	7%	19%
Molars *	31%	21%	16%

*—Statistically significant difference detected for this tooth type.

**Table 7:** Comparison of Agreement Between Diagnocat (DGNCT LLC, Miami, FL, USA) Analysis and Clinical Evaluation by Age Group and Tooth Type—Primary and Secondary Caries.

	18–34 Years (*n* = 57)	35–54 Years (*n* = 57)	55–68 Years (*n* = 13)	Test Result	*p*-Value	Percentage Structure Index Test (*p*-Value)		
	Agreement Between Diagnocat (DGNCT LLC, Miami, FL, USA) Analysis and Clinical Evaluation					1 vs. 2	1 vs. 3	2 vs. 3
Total	25.53%	15.58%	13.04%	14.07 a	0.0009	0.0007	0.0246	NS
Incisors	26.32%	9.76%	0.00%	7.78 b	0.0204	0.0541	0.0565	NS
Canines	25.00%	2.00%	0.00%	3.03 b	NS	NS	NS	NS
Premolars	13.73%	7.32%	19.05%	3.80 b	NS	NS	NS	0.0830
Molars	30.60%	20.92%	16.13%	6.90 b	0.0318	0.0232	0.0950	NS

Note: a—Pearson Chi-squared, b—Chi-squared NW, NS—Not significant.

**Table 8:** Comparison of Agreement Between Diagnocat (DGNCT LLC, Miami, FL, USA) Analysis and Clinical Evaluation for Selected Teeth Across Different Age Groups—Primary and Secondary Caries.

Tooth	18–34 Years (*n* = 57)	35–54 Years (*n* = 57)	55–68 Years (*n* = 13)	Test Result *	*p*-Value
	Agreement Between Diagnocat (DGNCT LLC, Miami, FL, USA) Analysis and Clinical Evaluation				
12	66.7%	22.2%	0.0%	4.90	0.0862
28	0.0%	66.7%	0.0%	5.18	0.0751
36	37.9%	5.9%	20.0%	6.79	0.0335
37	34.5%	20.0%	0.0%	5.02	0.0812
44	33.3%	0.0%	100.0%	7.96	0.0187

Note: *—Chi-squared NW.

**Table 9:** Agreement Between Diagnocat (DGNCT LLC, Miami, FL, USA) Analysis and Clinical Evaluation by Dentist for Different Types of Teeth—Primary and Secondary Caries.

	Dentist 1 (*n* = 49)	Dentist 2 (*n* = 48)	Dentist 3 (*n* = 30)
Incisors *	16%	8%	32%
Canines	6%	30%	31%
Premolars	7%	17%	11%
Molars *	18%	35%	28%

*—Statistically significant difference detected for this tooth type.

**Table 10:** Comparison of Agreement Between Diagnocat (DGNCT LLC, Miami, FL, USA) Analysis and Clinical Evaluation for Different Tooth Groups by Dentist—Primary and Secondary Caries.

	Dentist 1 (*n* = 49)	Dentist 2 (*n* = 48)	Dentist 3 (*n* = 30)	Test Result	*p*-Value	Percentage Structure Index Test (*p*-Value)		
	Agreement Between Diagnocat (DGNCT LLC, Miami, FL, USA) Analysis and Clinical Evaluation					1 vs. 2	1 vs. 3	2 vs. 3
Total	13.52%	25.38% *	23.72% *	16.00 a	0.0003	0.0002	0.0018	NS
Incisors	15.63%	7.69%	31.58%	5.21 b	0.0739	NS	NS	0.0183
Canines	6.25%	30.00%	30.77%	3.83 b	NS	NS	0.0821	NS
Premolars	7.08%	17.11%	10.53%	4.54 b	NS	0.0315	NS	NS
Molars	17.53%	34.53%	27.78%	12.81 a	0.0017	0.0004	0.0294	NS

Note: *—Significantly different from Dentist 1, a—Pearson Chi-squared, b—Chi-squared NW, NS—Not significant.

**Table 11:** Comparison of Agreement Between Diagnocat (DGNCT LLC, Miami, FL, USA) Analysis and Clinical Evaluation for Individual Teeth by Dentist—Primary and Secondary Caries.

Teeth	Dentist 1 (*n* = 49)	Dentist 2 (*n* = 48)	Dentist 3 (*n* = 30)	Test Result *	*p*-Value
	Agreement Between Diagnocat (DGNCT LLC, Miami, FL, USA) Analysis and Clinical Evaluation				
13	0%	20%	67%	9.26	0.0098
24	0.0%	15.4%	0.0%	4.61	0.0995
25	5.6%	40.0%	12.5%	5.23	0.0733
27	9%	50%	25%	9.20	0.0101
35	7%	13%	60%	6.7	0.0457
37	12.0%	38.9%	33.3%	4.72	0.0944
44	50%	0%	0%	6.24	0.0443
48	0%	75%	0%	10.94	0.0042

Note: *—Chi-squared NW.

**Table 12:** Agreement Between Diagnocat (DGNCT LLC, Miami, FL, USA) Analysis and Clinical Evaluation—Primary Caries in Women and Men.

	Men (*n* = 59)	Women (*n* = 68)
Incisors *	0%	26%
Canines	31%	20%
Premolars	12%	12%
Molars	38%	31%

*—Statistically significant difference detected for this tooth type.

**Table 13:** Comparison of Agreement Between Diagnocat (DGNCT LLC, Miami, FL, USA) Analysis and Clinical Evaluation in Detecting Primary Caries for Incisors, Considering Patient Gender.

Tooth	Men (*n* = 59) 46%	Women (*n* = 68) 54%	Test Result *	*p*-Value	*p*-Value
	Agreement Between Diagnocat (DGNCT LLC, Miami, FL, USA) Analysis and Clinical Evaluation				Percentage Structure Index Test
Incisors	0.00%	25.71%	5.17	0.0229	0.0082

Note: *—Pearson’s chi-square test with Yates’ correction.

**Table 14:** Agreement Between Diagnocat (DGNCT LLC, Miami, FL, USA) Analysis and Clinical Evaluation by Tooth Type and Patient Age Group—Primary Caries.

	18–34 Years (*n* = 57)	35–54 Years (*n* = 57)	55–68 Years (*n* = 13)
Incisors	24%	10%	0%
Canines	25%	36%	0%
Premolars	15%	8%	9%
Molars	37%	31%	9%

**Table 15:** Comparison of Agreement Between Diagnocat (DGNCT LLC, Miami, FL, USA) Analysis and Clinical Evaluation by Age Group and Tooth Type—Primary Caries.

	18–34 Years (*n* = 57)	35–54 Years (*n* = 57)	55–68 Years (*n* = 13)	Test Result	*p*-Value	Percentage Structure Index Test (*p*-Value)		
	Agreement Between Diagnocat (DGNCT LLC, Miami, FL, USA) Analysis and Clinical Evaluation					1 vs. 2	1 vs. 3	2 vs. 3
Total	28.85%	20.83%	5.71%	10.81 a	0.0045	0.0530	0.0034	0.0341
Incisors	24.14%	10.00%	0.00%	5.01 b	0.0819	NS	NS	NS
Canines	25.00%	36.36%	0.00%	2.98 b	NS	NS	NS	NS
Premolars	15.38%	8.33%	9.09%	NS	NS	NS	NS	NS
Molars	37.24%	31.46%	9.09%	4.69 b	0.0957	NS	0.0596	NS

Note: a—Pearson Chi-squared, b—Chi-squared NW, NS—Not significant.

**Table 16:** Comparison of Agreement Between Diagnocat (DGNCT LLC, Miami, FL, USA) Analysis and Clinical Evaluation for Selected Teeth Across Different Age Groups—Primary Caries.

Tooth	18–34 Years (*n* = 57)	35–54 Years (*n* = 57)	55–68 Years (*n* = 13)	Test Result *	*p*-Value
	Agreement Between Diagnocat (DGNCT LLC, Miami, FL, USA) Analysis and Clinical Evaluation				
13	0.0%	57.1%	0.0%	4.86	0.0880

Note: *—Chi-squared NW.

**Table 17:** Agreement Between Diagnocat (DGNCT LLC, Miami, FL, USA) Analysis and Clinical Evaluation by Dentist for Different Tooth Types—Primary Caries.

	Dentist 1 (*n* = 49)	Dentist 2 (*n* = 48)	Dentist 3 (*n* = 30)
Incisors *	7%	7%	46%
Canines *	0%	29%	50%
Premolars	8%	16%	13%
Molars *	21%	50%	32%

*—Statistically significant difference detected for this tooth type.

**Table 18:** Comparison of Agreement Between Diagnocat (DGNCT LLC, Miami, FL, USA) Analysis and Clinical Evaluation for Different Tooth Groups by Dentist—Primary Caries.

	Dentist 1 (*n* = 49)	Dentist 2 (*n* = 48)	Dentist 3 (*n* = 30)	Test Result	*p*-Value	Percentage Structure Index Test (*p*-Value)		
	Agreement Between Diagnocat (DGNCT LLC, Miami, FL, USA) Analysis and Clinical Evaluation					1 vs. 2	1 vs. 3	2 vs. 3
Total	14.21%	30.91%	28.78%	15.67 a	0.0004	0.0002	0.0013	NS
Incisors	6.67%	6.67%	46.15%	10.07 b	0.0065	NS	0.0161	0.0022
Canines	0.00%	28.57%	50.00%	6.94 b	0.0312	NS	0.0209	NS
Premolars	8.45%	16.00%	12.50%	1.63 b	NS	NS	NS	NS
Molars	21.35%	50.00%	32.05%	15.40 a	0.0005	0.0001	NS	0.0227

Note: a—Pearson Chi-squared, b—Chi-squared NW, NS—Not significant.

**Table 19:** Comparison of Agreement Between Diagnocat (DGNCT LLC, Miami, FL, USA) Analysis and Clinical Evaluation for Individual Teeth by Dentist—Primary Caries.

Tooth	Dentist 1 (*n* = 49)	Dentist 2 (*n* = 48)	Dentist 3 (*n* = 30)	Test Result *	*p*-Value
	Agreement Between Diagnocat (DGNCT LLC, Miami, FL, USA) Analysis and Clinical Evaluation				
13	0.00%	0.00%	80.00%	9.42	0.0090
27	12.50%	66.67%	27.27%	7.19	0.0275
35	10.00%	20.00%	75.00%	5.90	0.0524
48	0.00%	100.00%	0.00%	9.53	0.0085

Note: *—Chi-squared NW.

**Table 20:** Gender Distribution of Patients by Dentist—Bivariate Analysis.

Gender	Summary Bivariate Table: Observed Frequencies			
	Dentist 1	Dentist 2	Dentist 3	Total
Women	21	25	22	68
% of total	16.54%	19.69%	17.32%	53.54%
Men	28	23	8 *	59
% of total	22.05%	18.11%	6.30%	46.46%
Total	49	48	30	127
% of total	38.58%	37.80%	23.62%	100.00%

Note: * Chi^2^ = 7.01, *p* = 0.02999.

## Data Availability

Data are available on request due to privacy/ethical restrictions.
